# Dual-Dependency Attention Transformer for Fine-Grained Visual Classification

**DOI:** 10.3390/s24072337

**Published:** 2024-04-06

**Authors:** Shiyan Cui, Bin Hui

**Affiliations:** 1 Key Laboratory of Opto-Electronic Information Processing, Chinese Academy of Sciences, Shenyang 110016, China; cuishiyan@sia.cn; 2 Shenyang Institute of Automation, Chinese Academy of Sciences, Shenyang 110016, China; 3 Institutes for Robotics and Intelligent Manufacturing, Chinese Academy of Sciences, Shenyang 110169, China; 4 University of Chinese Academy of Sciences, Beijing 100049, China

**Keywords:** deep learning, fine-grained visual classification, vision transformer

## Abstract

Visual transformers (ViTs) are widely used in various visual tasks, such as fine-grained visual classification (FGVC). However, the self-attention mechanism, which is the core module of visual transformers, leads to quadratic computational and memory complexity. The sparse-attention and local-attention approaches currently used by most researchers are not suitable for FGVC tasks. These tasks require dense feature extraction and global dependency modeling. To address this challenge, we propose a dual-dependency attention transformer model. It decouples global token interactions into two paths. The first is a position-dependency attention pathway based on the intersection of two types of grouped attention. The second is a semantic dependency attention pathway based on dynamic central aggregation. This approach enhances the high-quality semantic modeling of discriminative cues while reducing the computational cost to linear computational complexity. In addition, we develop discriminative enhancement strategies. These strategies increase the sensitivity of high-confidence discriminative cue tracking with a knowledge-based representation approach. Experiments on three datasets, NABIRDS, CUB, and DOGS, show that the method is suitable for fine-grained image classification. It finds a balance between computational cost and performance.

## 1. Introduction

Fine-grained visual classification (FGVC) focuses on distinguishing different subclasses within the same metaclass, which is a fundamental task in computer vision and multimedia. Refs. [1,2,3,4,5] describe the related research progress that directly impacts the extension of a wide range of downstream task applications, such as image generation [6], fine-grained retrieval [7], and comparative learning [8]. In addition, FGVC methods are widely used in industrial and commercial applications such as biological protection [9,10], intelligent merchandising [11,12,13], and intelligent transportation [14,15].

**Figure 1:**
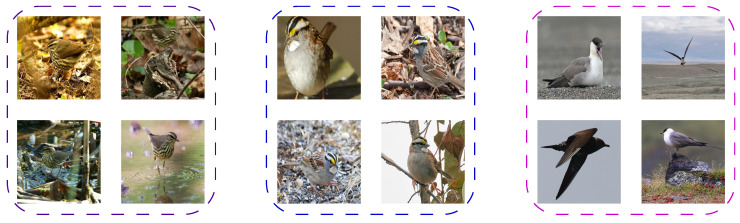
The images in the CUB-200-2011 dataset show that images surrounded by the same dashed box represent the same category, and it is intuitively clear that achieving fine-grained visual classification is challenging.

In contrast to coarse-grained classification, the extraction of discriminative information for fine-grained classification requires attention to subtle gaps in features and the modeling of complex relationships between discriminative cues. Figure 1 illustrates that FGVC has a distinct characteristic of small interclass similarity and large intraclass variability due to the impact of the imaging environment, including the object’s pose, light changes, and viewpoint rotation; thus, fine-grained classification is challenging to achieve.

Previous studies [16,17,18] used different combinations of location and category labels, but the specificity of FGVC is the threshold of expertise in data labeling. As a result, some studies adopted a weakly supervised approach [19,20,21,22] by adding a manually designed module to realize the ability of model region selection, but this usually involves a more complex training process. In later studies [19,20,21,22], an attentional learning mechanism was used to adaptively learn highly responsive representations for important features, allowing end-to-end training. The success of Vision Transformer in most visual tasks has led to research into fine-grained visual classification, where the methods [23,24,25,26,27,28] involve the use of a powerful self-attention mechanism to guide the localization of important feature regions, combining the expression of backbone features with refined learning of local features to outperform previous methods. However, the computational cost of the self-attention mechanism used by the model is positively correlated with the square of the image resolution. There is a lack of self-attention mechanisms with linear computational complexity that can be used to directly replace the original version without targeted pre-training.

Analyzing the central role of ViT-based methods in fine-grained visual classification is crucial. The ability to model long-range dependencies on feature tokens is an important foundation. It allows models to identify key feature regions and establish discriminative feature extraction strategies. However, the interaction of global tokens significantly increases computational complexity. Unfortunately, attention methods with linear computational complexity, such as sparse attention and windowed attention, are not suitable for fine-grained visual tasks. While they are suitable for coarse-grained visual tasks, they do not meet the feature learning requirements of fine-grained challenges. These methods result in a lack of detailed features, leading to incomplete discriminative cues. In addition, they cut off the learning of dependencies between discriminative cues, leading to a deterioration in discriminative ability.

To address the above issues, we introduce the Dual-Dependency Attention Transformer (DDA-Trans), which transforms the original global token interaction into a dual-pathway interaction. It combines local dense attention and global sparse attention in the position dependency pathway and exploits the self-renewal and information propagation of center tokens with data-specific aggregation in the semantic dependency pathway. Through this dual-dependency modeling, the model reduces redundant interactions and improves the quality of feature representation. It also strengthens the ability to identify cues associated with high-confidence categories, with improvement achieved through knowledge-based discriminative ability enhancement learning.

Our model starts by segmenting input image features, combined with position encoding, into non-overlapping patches and then transforming these patches into a linear token sequence. To model the connection between features and their aggregation centers, we use a feature center aggregation (CTA) learning module, which allows it to capture variations in data distribution and ensures the data specificity of center token generation. In our backbone network, we replace the initial attention mechanism with dual-dependency attention (DDA). The position-dependency attention (PDA) pathway of DDA employs window splitting and in-window interaction for global tokens. This is achieved by cross-using two window-splitting methods: local dense attention (LDA) windows and global sparse attention (GSA) windows. In the semantic-dependency attention pathway (SDA), the clustering process of the center token acts as a mediator for information exchange between global tokens. The information transfer direction involves the center token extracting global tokens for self-renewal and the global tokens extracting the center token for self-interaction. The global interaction is based on the decoupling of global features in the semantic space by the aggregation of the center token, which realizes the dependency modeling between tokens with similar semantic information. In addition, our model employs the Discriminative Ability Enhancement (DAE) module to supplement the knowledge of high-confidence categories of cls tokens. This is achieved through knowledge modeling for the classification target, which strengthens the discriminative feature extraction capability of the model. Combined with progressive knowledge guidance, the model outputs the final discrimination, striking a balance between computational efficiency and detailed feature extraction in fine-grained visual classification tasks.

Our contribution can be summarized as follows:

- We propose a dual-dependency attention mechanism with a linearly positive correlation of computational complexity, which can be realized instead of the original attention to be directly fine-tuned in FGVC without the need for pre-training for the new attention.
- We propose a knowledge-based discriminant ability enhancement method to improve the sensitivity to the corresponding cues of high-response categories.
- We validate the models on multiple datasets and perform interpretable analyses of the model learning mechanisms using the visualization results.

## 2. Related Work

### 2.1. CNN-Based Model for FGVC

In 1989, LeCun et al. [29] proposed CNNs that achieved excellent results in some computer vision tasks, after which a large number of classical models such as AlexNet [30] and GoogleNet [31] were also proposed, extending the application of CNN models to more visual downstream tasks, such as fine-grained visual classification. The models map image features to category confidence to complete classification via deep convolutional neural networks and fully connected networks. The application research in FGVC can be divided into two categories.

#### 2.1.1. Component Localization Method

This method usually achieves fine-grained feature extraction by localizing important discriminative features. Huang et al. [32] proposed to improve the discriminative ability using foreground target localization labels added to the training of the model. Liu et al. [33] designed a weakly supervised cross-part convolutional neural network for localizing multi-region features and learning cross-part features. Yang et al. [34] used a combination of coarse and fine class prediction to localize the area with the help of coarse classification and then used fine classification for discrimination. Ge et al. [35] proposed combining Mask R-CNN networks for the segmentation of locally important regions, and He et al. [36] used deep reinforcement learning methods to train strategy models, which are usually accompanied by complex network structures, for regional feature screening.

#### 2.1.2. Attention Screening Method

Instead of manually designed region localization, this approach focuses on adaptive discriminative feature capture through attention learning. Zheng et al. [37] achieved component discrimination by applying attention to the feature channel. Ding et al. [20] used the response differences of the attention map to realize the determination of critical regions for foreground targets. Zhuang et al. [38] proposed a cross-attention–guided model for contrast learning on paired images to enhance the capture ability of the model for discriminative cues. Zhao et al. [3] used the attention mechanism to achieve high-quality feature characterization of images combining multi-scale and multi-granularity features. Luo et al. [39] designed attention modeling between different images and between different network layers to improve the robustness of multi-scale feature learning.

### 2.2. ViT-Based Model for FGVC

Transformer [40,41] has achieved remarkable success in the field of natural language due to its powerful learning ability through self-attention mechanisms, and in light of this exceptional performance, researchers have projected their interest in studying its application in the field of computer vision. Dosovitskiy et al. proposed that ViT [42] shows strong performance in a variety of basic computer vision tasks, such as image classification, target detection, and image segmentation, where the model uses the main structure of Transformer while segmenting the image features into patches of tokens with linear sequences and encoding the absolute positional relationships between the tokens. ViT enables the modeling of global feature dependencies, making it powerful for fine-grained feature extraction and discriminative cue construction.

Most ViT-based models [43] inherit the local region paradigm; He et al. [44] removed noise interference by suppressing the expression of image background features in discrimination using attention maps, while Xu et al. [24] and Wang et al. [45] focused on using attention to filter cross-layer features, combining the weights of the feature layer and the attention head for fusion learning in the final layer. Sun et al. [25] used simple graph networks for fusion and refinement of highly responsive cross-layer tokens and enhanced model robustness with contrast learning. Liu et al. [46] proposed to combine the suppression of the highest response token for secondary learning of image features to enhance the richness of the refined feature extraction. Hu et al. [28] used a dual-backbone network to process global features and localized area refinement features separately, and Zhu et al. [27] proposed dual–cross-attentional learning focusing on the interaction between global and local features and cross-learning between pairs of images, where the local region is selected according to the global backbone guidance.

### 2.3. Vision Transformer Acceleration

Despite the tremendous impact of ViT in computer vision, the computational cost grows quadratically with the image size, which is challenging for applications in downstream tasks. Most recent studies have tended to assume a priori that image features are sparse and localized to create an inductive bias in the design of the model structure.

Some ways to learn using sparse attention are as follows: Wang et al. [47] introduced a feature pyramid structure using convolution to reduce the spatial dimension and perform feature downsampling. Zeng et al. [48] proposed to transform the generation of tokens into a dynamic merger by incrementally aggregating them. Yang et al. [49] used localized attention with the introduction of convolution in the low-level feature stage and focused on multi-scale contextual features in the high-level feature stage. Another approach focused on limiting the spatial range of attention learning. Liu et al. [50] realized the interaction between different window tokens by combining moving windows with window attention, and Yang et al. [51] used sampling of multiple steps as the key and value of tokens for localized attention to achieve acquisition of attention learning at multiple scales. Finally, Tu et al. [52] used a combination of local block attention and dilated global attention that allows global–local spatial interactions at arbitrary input resolutions.

While these approaches retain the ability to allow each token to focus indirectly on global image tokens, the semantic description of low-fidelity tokens leads to a lack of fine-grained feature representations and inefficient modeling of dependencies between key discriminative cues. Our approach learns feature space dependencies while focusing on modeling semantic information related to the subject elements of the image and achieves adaptive grouping of dependencies on key discriminative semantic information according to the aggregation center in the feature semantic space, which can be used to meet the need for high-quality feature tokens.

## 3. Method

**Figure 2:**
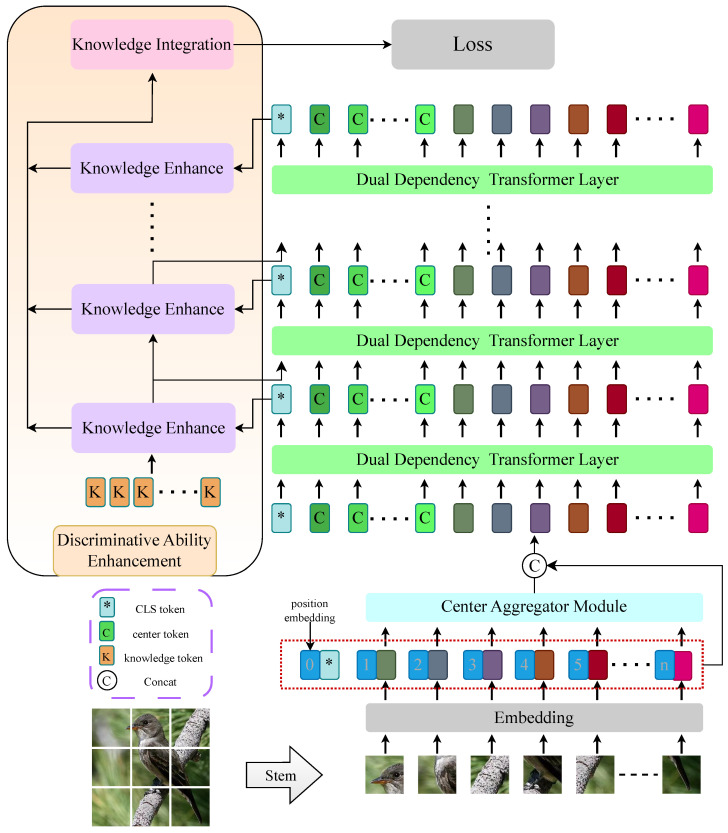
Overall structure of the model. The global token input center aggregation module (CTA) obtains the center tokens, combines and feeds them into the backbone network with dual-dependency attention (DDA), and connects the cls tokens of each layer to the discriminative ability enhancement module (DAE).

We propose DDA-Trans for fine-grained visual classification, and the whole structure is shown in Figure 2.

### 3.1. Original Vision Transformer

ViT processes a 2D image into a 1D sequence, similar to the string format commonly used in NLP, and then feeds it to an encoder stacked by transformer layers, with the core structure within the transformer layers being the self-attention mechanism, which allows the model to realize global feature dependency modeling with data specificity.

The computational process of the self-attention mechanism can be described as mapping features into query vectors, key vectors, and value vectors, obtaining global attention by computing the dot product between query vectors and key vectors, considering the attention weights after scaling and normalization as weighted weights of the value vectors, and computing the weighted results to obtain the new feature representation. Specifically, $Query,Q\in R^{N\times D}$, $Key,K\in R^{N\times D}$, and $Value,V\in R^{N\times D}$ are obtained by feature mapping of input $X\in R^{N\times D}$, and the mapping matrices are $W_{q}\in R^{D\times D}$, $W_{k}\in R^{D\times D}$, and $W_{v}\in R^{D\times D}$, where *c* is the number of channels in the feature and *N* is the number of tokens obtained in the original image. For any query vector $q\in R^{1\times D}$, it is necessary to interact with all key vectors; then, the complete self-attention computation can be represented as follows:

(1) $$Atten\left(Q,K,V\right)=Softmax\left(\frac{QK^{T}}{\sqrt{D}}\right)V$$

where $\sqrt{D}$ is a scaling factor.

Based on self-attention, feature channels are assigned to multiple heads within separate self-attention computations for multi-head self-attention (MSA). The feedforward network (FFN) has two fully connected layers with residual connections. A transformer encoder block can be constructed using the MSA layer and the FFN layer. The forward propagation of the *k*-th layer is calculated as follows:

(2) $$z_{k}^{*}=LN\left(MSA\left(z_{k-1}\right)+z_{k-1}\right)$$

(3) $$z_{k}=LN\left(FFN\left(z_{k}^{*}\right)+z_{k}^{*}\right)$$

where LN(·) indicates the Layer Normalization operation [53], k=1,2,3…L, and *L* is the number of layers.

### 3.2. Center Token Aggregation Module

The guiding role of the center feature in dual-dependency attention (DDA) is crucial for determining the semantic window construction of the semantic-dependency attention pathway (SDA). The effective play of DDA in each layer of the model largely depends on the ability of the center token feature to capture the key information. Due to the specificity of data distribution in fine-grained visual classification, the aggregation method of the center token needs to be dynamically adjusted according to the specificity of the data. Therefore, traditional static weighting methods may not be applicable in this case, and the model needs to explore more flexible aggregation strategies. This is to enable the model to adapt to the dynamic nature of fine-grained data and to fully exploit the information of the key features.

**Figure 3:**
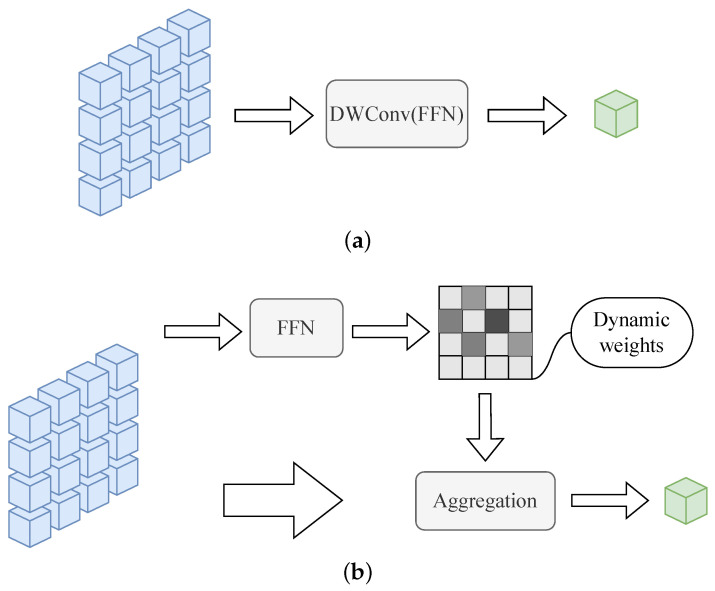
Center aggregation module structure diagram. The figure shows the difference between our adaptive dynamic aggregation (**b**) and commonly used static aggregation methods (**a**).

To address the above problems, we design the central aggregation (CTA) approach shown in Figure 3 to pay full attention to global features as a prerequisite, transform the commonly used static aggregation approach into self-directed dynamic aggregation, and learn the mapping from feature description to aggregation weights. The efficient aggregation approach guarantees that the center token realizes the complete inheritance of semantic representations of foreground targets and background details, avoiding discrimination-irrelevant semantic dependencies in the subsequent attention learning.

Specifically, we obtain the feature token $X_{P}\in R^{N\times D}$ with positional embedding, where *N* is the number of patch tokens, feed it into the fully connected network to obtain the token weights, and use the normalized weights to weight the global token to obtain the center token; the mapping process can be expressed as follows:

(4) $${X^{\prime }}_{P}=ACT\left(LN\left(X_{P}\right)W_{1}\right)$$

(5) $$X_{S}=ACT\left({X^{\prime }}_{P}W_{2}\right)$$

where *ACT* is the GELU, $W_{1}\in R^{D\times \frac{D}{4}}$ and $W_{2}\in R^{\frac{D}{4}}\times m$ are the learnable parameter, *m* is the hyperparameter as center token number, LN(·) indicates the Layer Normalization operation [53], and $X_{S}\in R^{N\times m}$ is the token weight. It can then be obtained as follows:

(6) $$X_{C}=Softmax\left(X_{S}^{T}\right)X_{P}$$

where $X_{S}^{T}$ denotes the transpose of $X_{S}$ and $X_{C}\in R^{m\times D}$ is the center token.

### 3.3. Dual-Dependency Attention

**Figure 4:**
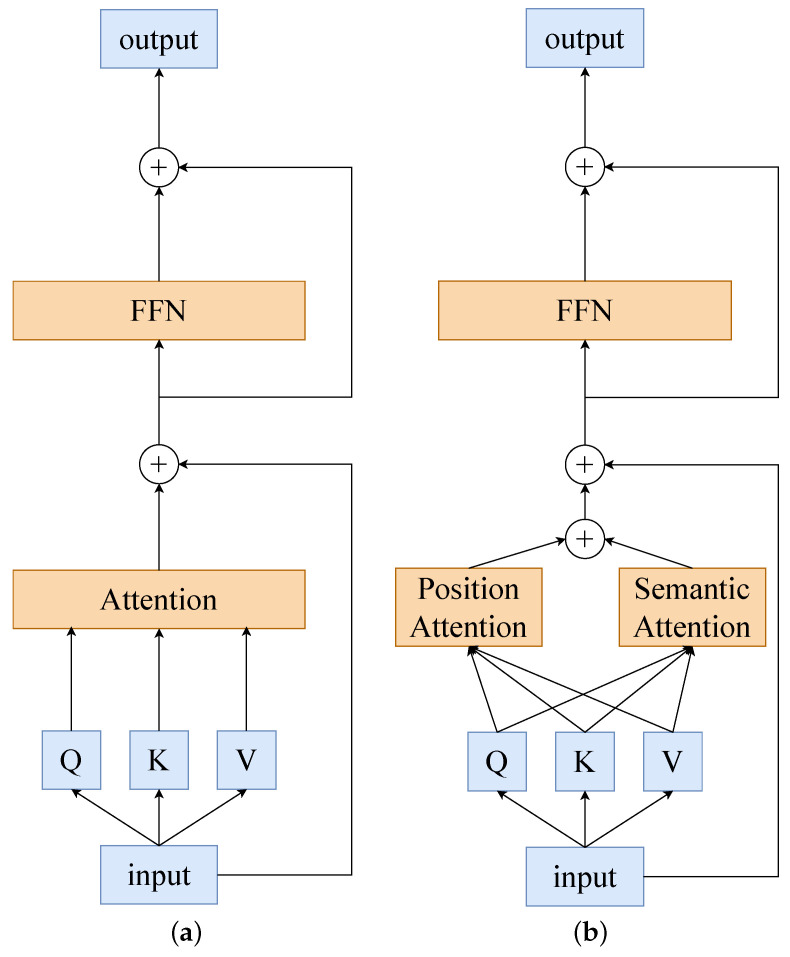
The figure illustrates our dual-dependency learning transformer layer structure. In contrast to the original vision transformer layer, we decompose the attention module into a positional spatial attention pathway and a semantic spatial attention pathway, whose computational cost is linearly related to the number of tokens; (**a**) Original vision transformer layer; (**b**) Dual-dependency transformer layer.

The original self-attention mechanism allows each token to interact with all tokens to form a global token dependency and to address its huge computational cost. We decouple it into a dual-dependency learning in position space (PDA) and semantic space (SDA), which achieves an alternative to the original method with a linear positive correlation computational cost. In Figure 4, a comparison of our novel attention with the original method can be found.

**Figure 5:**
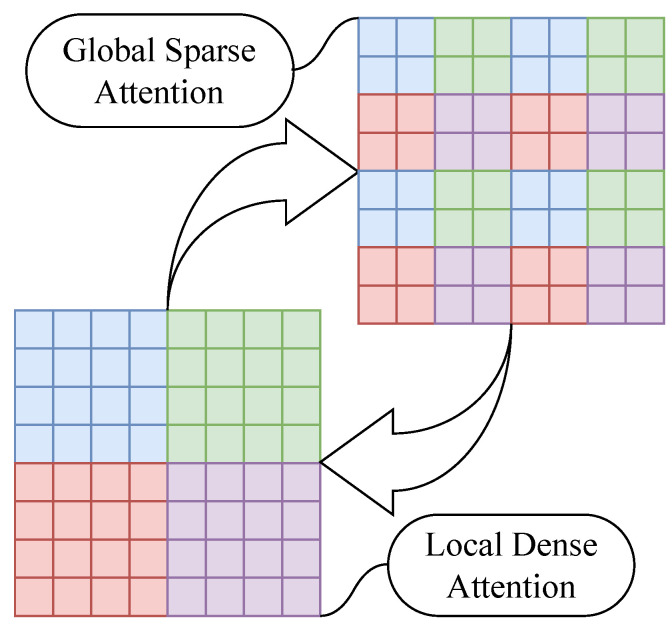
Schematic of token interactions for position space attention pathways. Local dense attention and global sparse attention cross out in the position space pathways at each layer of the backbone network. Tokens with the same color are grouped into the same groups for interaction.

In the position space pathway (PDA), we design two types of grouping token interactions, namely, local dense attention (LDA) and global sparse attention (GSA), and these interactions are shown in Figure 5. In LDA, tokens are grouped according to their relative positions in space by windowing, and each token only needs to interact with all tokens within its window. In GSA, all tokens within each window are grouped again so that each new group contains tokens from each window, and the scope of token interactions is restricted to the group. We design to allow these two types of attention to stack up in the backbone network, making the model perform global token interactions within two neighboring layers.

Specifically, we obtain the feature map $X\in R^{N\times D}$ and determine the number *g* of tokens within each group, its shape transforms to the same $X\in R^{g\times \frac{N}{g}\times D}$ when the feature map is fed into local dense interactions and global sparse interactions in the location-space pathway. In addition, to enhance the information interaction between intragroup tokens and global tokens, the keys and values of cls tokens and center tokens are shared within each group; therefore, we obtain $Query,Q_{P}\in R^{g\times \frac{N}{g}\times D}$, $Key,K_{P}\in R^{g\times O\times D}$, and $Value,V_{P}\in R^{g\times O\times D}$, where $O=\frac{N}{g}+m+1$, and the positional dependency attention is calculated as follows:

(7) $$Atten_{P}\left(Q_{P},K_{P},V_{P}\right)=Softmax\left(\frac{Q_{P}K_{P}^{T}}{\sqrt{D}}\right)V_{P}$$

It is worth noting that although LDA and GSA are consistent in the computational process, they differ significantly in the windowing strategy of feature tokens. Specifically, in the LDA, the feature space is directly divided equally according to a predetermined number of windows, such that feature tokens belonging to the same window are spatially adjacent. This partitioning facilitates the capture of subtle feature changes in the local region, which is conducive to increasing the sensitivity to local structures. In contrast, the GSA uses a more globalized window partitioning strategy that achieves global feature fusion by uniformly distributing the tokens within each local window to each global window. This strategy allows each global window to absorb information from different local regions, enhancing the ability to understand the global structure and contextual information.

In the semantic space pathway (SDA), global tokens interact semantically by extracting information from the center token, and the center token updates itself by extracting information from the global tokens. The center token serves as a medium for information propagation, allowing each token to interact with the global token with linear computational complexity. It is worth noting that this dependency modeling is based on the cross-attention relationship between the center token and the global token, which means that the method of information propagation depends on the distribution of the center token in the semantic space of the entire image features. In other words, our proposed semantic space attention can be viewed as an overlapping partition of the image semantic space based on the center token, and the dependency modeling of the semantic information in the partitioned subspace guarantees a high-quality feature representation of the semantics of the image object. Furthermore, the demonstration of the visualization results for the model principle supports this assertion.

**Figure 6:**
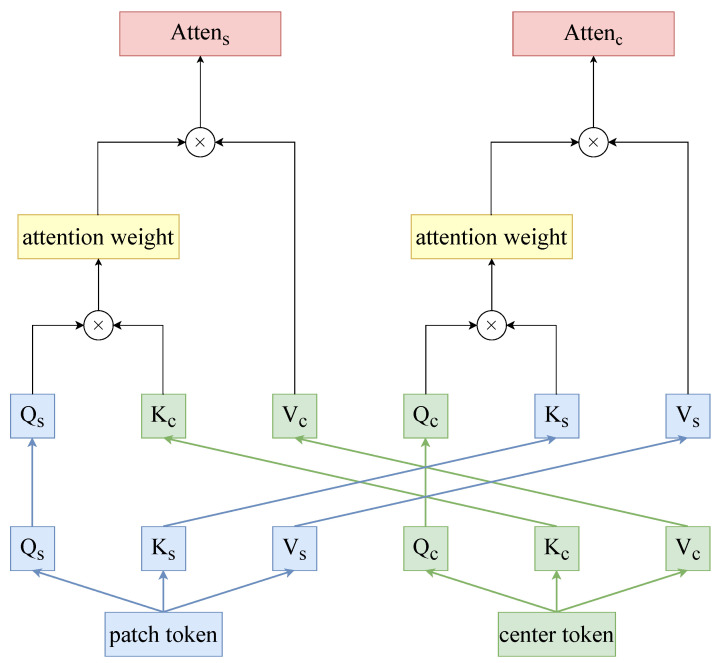
Diagram showing the structure of the semantic-dependency attention pathway. Cross-attention between global tokens and central tokens can be implemented with linear computational complexity for interactions between global tokens.

Specifically, we use cross-attention learning of center tokens with global tokens to achieve semantically relevant interactions of global tokens and clustering updates of center tokens, with the structure shown in Figure 6. We take the global tokens $X\in R^{N\times D}$ and the center tokens $X_{C}\in R^{m\times D}$; after feature mapping, we obtain $Q_{S}\in R^{N\times D}$, $K_{S}\in R^{N\times D}$, and $V_{S}\in R^{N\times D}$ and $Q_{C}\in R^{m\times D}$, $K_{C}\in R^{m\times D}$, and $V_{C}\in R^{m\times D}$, and the semantic-dependency attention is calculated as follows:

(8) $$Atten_{S}\left(Q_{S},K_{C},V_{C}\right)=Softmax\left(\frac{Q_{S}K_{C}^{T}}{\sqrt{D}}\right)V_{C}$$

(9) $$Atten_{C}\left(Q_{C},K_{S},V_{S}\right)=Softmax\left(\frac{Q_{C}K_{S}^{T}}{\sqrt{D}}\right)V_{S}$$

It is interesting to note that in the SDA, we do not perform center token interaction (CI). The purpose of this design is to ensure that the center token can serve as the core of the information transfer, preserving as much as possible the discreteness of interest of each semantic window. This discreteness is crucial because it ensures that the semantic information of the subject instances in the global image feature can be learned in its entirety. By maintaining the independence of the central token, the model gains an enhanced ability to recognize critical details in the image by effectively freeing it from unnecessary information obfuscation.

Otherwise, the generation of the cls token has not changed; we take $X\in R^{N\times D}$ and $X_{cls}\in R^{1\times D}$ to form $Q_{cls}\in R^{1\times D}$, $K\in R^{N\times D}$, $V\in R^{N\times D}$, $K_{cls}\in R^{1\times D}$, and $V_{cls}\in R^{1\times D}$, and the new cls token is calculated as follows:

(10) $$\begin{array}{c} K_{all}=concat\left(K,K_{cls}\right) \end{array}$$

(11) $$\begin{array}{c} V_{all}=concat\left(V,V_{cls}\right) \end{array}$$

(12) $$\begin{array}{c} Y_{cls}=Softmax\left(\frac{Q_{cls}K_{all}^{T}}{\sqrt{D}}\right)V_{all} \end{array}$$

where $Y_{cls}\in R^{1\times D}$ is the output of the cls token in the dual-dependency attention.

After the dual-dependency attention, we obtain two feature representations for the global tokens, and each token needs to be evaluated for its weight in both pathways. Specifically, in the semantic-dependency attention pathway, we consider that the significant tendency of patch tokens to pay attention to all center tokens indicates that their semantic information is more relevant to the image subject, so we compute the variance of the attentional weights of each patch token for all center tokens and map it to $\left[0.5,1.5\right]$ and expand the dimension of the weight vector as $W\in R^{N\times D}$; the fusion is then calculated as follows:

(13) $$Y=Atten_{P}+Atten_{S}⊗W\times \alpha$$

(14) $$Y_{C}=X_{C}+Atten_{C}\times \beta$$

where ⊗ implies element-wise multiplication, α and β are two learnable parameters that are used as scaling factors, and $Y\in R^{N\times D}$ and $Y_{C}\in R^{m\times D}$ are the output of global tokens and center tokens in the dual-dependency attention.

### 3.4. Complexity Analysis for Dual-Dependency Attention

In the field of fine-grained image classification, the Vision Transformer has been studied due to its computational cost, which is quadratically positively correlated with the resolution of the input image; however, there is a lack of substitutes that can be used for the linear complexity attention of the FGVC without the need for targeted pre-training. In the following, we compare the computational complexity of our dual-dependency attention with the standard global self-attention.

For the global self-attention, query mapping, key mapping, value mapping, self-attentive learning, and output mapping are required for the input feature map $X\in R^{N\times D}$, where *N* is the number of patch tokens and cls tokens; the global self-attention can be expressed as follows:

(15) $$\begin{array}{c} Q=query(X)\;K=key(X)\;V=value(X) \end{array}$$

(16) $$\begin{array}{c} A=Softmax(\frac{QK^{T}}{\sqrt{D}})V \end{array}$$

(17) $$\begin{array}{c} O=output(A) \end{array}$$

where the corresponding computational complexity can be expressed as follows:

(18) $$O\left(GSA\right)=2N^{2}D+4ND^{2}$$

where it is obvious that the original global attention approach has a significant computational cost.

For our dual-dependency attention, the method is the same as the original method regarding query mapping, key mapping, value mapping, and output mapping, so we mainly analyze the attention calculation process.

In the position space–dependency attention pathway, attention is computed with the feature map $X\in R^{g\times \frac{N}{g}\times D}$ as follows:

(19) $$A_{pda}=Softmax\left(\frac{Q_{P}K_{P}^{T}}{\sqrt{D}}\right)V_{P}$$

where the corresponding computational complexity can be expressed as follows:

(20) O(pda)=2gND+2mND

In the semantic space–dependency attention pathway, attention is computed with the feature map $X\in R^{N\times D}$ and $X_{C}\in R^{m\times D}$ as follows:

(21) $$A_{sda_{s}}=Softmax\left(\frac{Q_{S}K_{C}^{T}}{\sqrt{D}}\right)V_{C}$$

(22) $$A_{sda_{c}}=Softmax\left(\frac{Q_{C}K_{S}^{T}}{\sqrt{D}}\right)V_{S}$$

where the corresponding computational complexity can be expressed as follows:

(23) O(sda)=2mND+2mND=4mND

Thus, the computational complexity of our dual-dependency attention can be expressed as follows:

(24) $$\begin{array}{cc} O(DDA) & =O\left(pda\right)+O\left(sda\right)+4ND^{2} \end{array}$$

(25) $$\begin{array}{cc} O(DDA) & =2gND+6mND+4ND^{2} \end{array}$$

It is clear that the computational cost of our proposed dual-dependency attention has only a linear positive correlation with the input image resolution and that *g* and *m* are much smaller than *N*, implying significant computational reductions.

### 3.5. Discriminative Ability Enhancement Module

**Figure 7:**
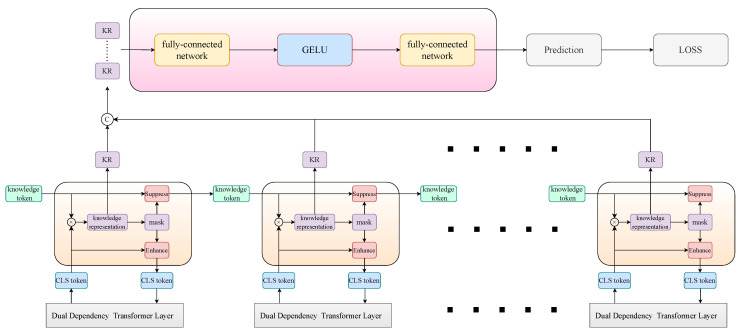
Diagram showing the structure of semantic-dependency attention pathway. Cross-attention between global tokens and central tokens can be implemented with linear computational complexity for interactions between global tokens.

The detailed structure of the module is shown in Figure 7. The discriminative accuracy of the model depends on the sensitivity of the fine-grained feature extraction strategy to the discriminatively relevant refinement information in the feature space. Based on our dual-attention–dependent high-fidelity feature representation, our proposed discriminative ability enhancement module implements guidance for the tendency change in the fine-grained feature extraction strategy in the backbone network by taking advantage of the knowledge-based modeling of classification recognition cues.

Observation of the classification results of the model shows that in most misclassification cases, the confidence level of the correct category is usually only slightly lower than the highest confidence level, suggesting that these misclassification cases are caused by insufficient extraction of relevant discriminative features for a small number of categories. Therefore, we decided to strengthen the ability of the model to capture discriminative cues for high-confidence classes.

Specifically, we designed serial-connected knowledge enhancement learning to connect with each layer in the backbone network and augment the cls token output from each layer with discriminative knowledge. The model suppresses high-confidence class representations of knowledge tokens to guarantee the richness of fine-grained feature extraction for cls tokens and transfer and fuse the knowledge representation of the cls tokens. In single knowledge enhancement learning, we take the knowledge token $X_{K}\in R^{k\times D}$ and the cls token $X_{cls}\in R^{1\times D}$, where *k* is the number of classes, and the knowledge representation $s\in R^{1\times K}$ corresponding to the cls token can be computed in the following:

(26) $$s=Softmax(\frac{X_{cls}X_{K}^{T}}{\sqrt{D}})$$

Based on the sorting of the knowledge representation according to the confidence value, we obtain the mask $M_{s}\in R^{1\times K}$ corresponding to the top *t* classes and the mask $M_{n}\in R^{1\times K}$ corresponding to the other classes, and the output is computed as follows:

(27) $$\begin{array}{c} X_{cls}=X_{cls}+M_{s}X_{K}\times \gamma \end{array}$$

(28) $$\begin{array}{c} X_{K}=M_{s}⊗X_{K}\times \epsilon +M_{n}⊗X_{K} \end{array}$$

where ⊗ implies element-wise multiplication with broadcasting and γ and ϵ are two learnable parameters that are used as scaling factors. It accurately assesses the deflection of the model discrimination results by the knowledge enhancement module and absorbs the multi-level fine-grained features to exploit the learning richness of the model features. The knowledge representation *s* of each layer of the cls tokens is spliced into $S\in R^{L\times K}$, where *L* is the number of backbone layers, and the final discrimination $P\in R^{1\times K}$ of the model is obtained by employing the learnable knowledge integration method, which is calculated as follows:

(29) $$P=W_{4}ACT\left(W_{3}S\right)$$

where ACT is the GELU, $W_{3}\in R^{L\times L}$ and $W_{2}\in R^{1\times L}$ are the learnable parameter, and *P* is the output of the model.

## 4. Experiments

In this section, we describe our experiments and discuss the results. We first show three datasets with the experimental setup, and then we show the specific experimental results for each of our datasets separately and compare them with state-of-the-art methods as well as detailed ablation experiments on the structure of our network, which delve into the specific effects of each component. In addition, we show the visualization results used for the interpretability analysis of the model, which intuitively illustrates how the model works.

### 4.1. Datasets

Three benchmark datasets are used in our experiments, namely, CUB-200-2011 [54], Stanford Dogs [55], and NABirds [56]. According to the content of the datasets, CUB-200-2011 and NABirds are fine-grained datasets for bird classification, and Stanford Dogs contains images of dogs from all over the world. In terms of dataset size, CUB-200-2011 and Stanford Dogs are medium-sized datasets, while NABirds is a large dataset. Detailed information on these datasets is given in Table 1. The images in these three datasets are divided into two groups that are the training set and the test set, and the two groups of images in each dataset contain a similar number of images with detailed annotations of component position coordinates and bounding boxes, but it is worth noting that our approach uses only class labels.

To quantitatively evaluate the effectiveness of our method, the classification accuracy is obtained and calculated as follows:

(30) $$Accuracy=\frac{TP}{TP+FP}$$

where *TP* indicates true-positive test results, which are positive and correct, and *FP* indicates false-positive test results, which are negative but have been misinterpreted as positive.

### 4.2. Implementation Details

ViT-B-16 pre-trained on ImageNet21K is used as the backbone network, the input image size is 448×448, we use data augmentation including random cropping and horizontal flipping during training, and only center cropping is used in testing. The model was trained using a stochastic gradient descent (SGD) optimizer with a batch size of 32 and a momentum of 0.9 for all datasets. The learning rate was initially set to $2\times 10^{-2}$, and the scheduling applied the cosine decay function to the optimizer. The model was trained for 50 epochs, and the DAE module was not used for the first 10 epochs, as the basic formation of the class discriminative knowledge modeling is required for the module to achieve discrimination improvement. In addition, our model was implemented in PyTorch on Nvidia GeForce RTX 3090 GPUs, and the evaluation metric for all experiments was top-1 accuracy.

### 4.3. Ablation Experiments and Analysis

To verify that each component of our dual-dependency learning transformer effectively improves the classification precision for fine-grained images, we performed ablation studies and analyses. In order to verify the validity of all the improvement schemes in our model, ablation experiments were performed on several modules of the model, and the results are shown in Table 2. The ✓ indicates that the module is used and the ✕ indicates that it is not used, which applies to all Table.

The modules involve center token aggregation (CTA), position-dependency attention (PDA), semantic-dependency attention (SDA), and discriminative ability enhancement (DAE). Observing the experimental results, we can intuitively find that our dual pathway attention learning method is complementary to performance enhancement, and the cooperation between the two will always achieve higher performance than either of them alone; moreover, our novel semantic-dependency attention pathway based on dynamic aggregation always plays the most important role in the feature modeling process; in addition, our discriminative ability enhancement module makes a significant contribution to improving the classification accuracy of the model.

Since our approach involves the choice of the hyperparameter *m* the number of center tokens, *g* the number of tokens in a group, and *t* the number of high-confidence classes selected, we experimentally obtain the effect on the classification performance, which is summarised in Table 3. It is worth noting that although a larger number of in-group tokens may provide a performance gain, we balance the improved performance against the computational cost required, making m=16,g=48,t=8 our choice. It is worth noting that we obtain the initial choice of each hyperparameter m=16,g=48,t=8 after the search and check whether there is any mutual influence between hyperparameters through control variables, respectively, and the experiment shows that they do not interfere with each other. Finally, the model employs m=16,g=48,t=8.

In order to verify whether our proposed dynamic aggregation method (CTA) for center token aggregation generation is superior to the traditional static aggregation method (TSA), the experimental results are shown in Table 4. As a result, it is clear that regardless of the dataset, the dynamic feature-based adaptive aggregation approach always provides a more data-specific center token initialization for the semantic-dependency attention pathway to facilitate the iterative updating of the center tokens and the semantic-dependency modeling of the global tokens.

In our position-dependency attention pathway, the two types of grouped attention, local dense attention (LDA) and global sparse attention (GSA), are present in the backbone network layer in a cross-construction situation, for which the ablation experiments are recorded in Table 5. Although they have the same amount of computation, the cross-construction of the two grouping attention is clearly more favorable to the indirect interaction of global tokens, which can yield a performance gain.

In addition, in our semantic-dependency attention pathway, we did not perform any interaction learning between center tokens. The relevance interaction learning in the semantic space requires that the clustering centers can capture a complete representation of the global semantics, while a certain degree of independence between the centers is required to ensure the effective modeling of the semantic dependencies. The experimental results in Table 6 support this viewpoint, and the model can achieve better performance without the center token interactions.

### 4.4. Comparison to Other SOTA Methods

For the sake of fairness, we eliminate the overlapping split token generation method and the various training labels except for the class labels for all ViT-based models.

#### 4.4.1. Experiments on the CUB-200-2011 Dataset

Comparison of the SOTA methods at this stage, as shown in Table 7. Our proposed DDA-Trans model achieves an improvement of 1.1% over the best-performing CNN-based model CAL [57] and 0.9% over the ViT [42], showing that our method still has strong performance while achieving a large reduction in computational cost. However, compared to the state-of-the-art ViT-based method IELT [24], which uses ViT as the backbone network while adding a transformer layer, our method has only a performance gap of 0.1% with fewer parameters and computational cost, which shows that our linear computational complexity of attention does not limit the ability of fine-grained feature extraction and also proves that our attention mechanism is a highly efficient and low-cost method for FGVC.

#### 4.4.2. Experiments on the Stanford Dogs Dataset

The state-of-the-art methods at this stage are organized in Table 8. We can see that our model obtains 1.7% and 1.0% improvements compared to the state-of-the-art CNN-based model PRIS [66] and ViT [42], respectively, which are higher than the results for CUB-200-2011, indicating that our method does not fail due to the increase in the amount of data. However, compared to the state-of-the-art ViT-based method TPSKG [46], which uses dual backbone network forward propagation during the training process, resulting in a significant increase in computational complexity during the training phase, our method has only a performance gap of 0.1% with less computational complexity and achieves the same performance as the RAMS-Trans [28] method, which uses dual backbone networks for both training and inference. Clearly, the effectiveness of our method is once again confirmed.

#### 4.4.3. Experiments on the NABirds Dataset

According to Table 9 comparing the state-of-the-art methods, we can see that our model obtains 2.2% boosting compared to the state-of-the-art CNN-based model MGE-CNN [67] and 0.9% boosting compared to the ViT [42], which indicates that our method does not lose model capacity due to the reduction in computational cost. Compared to the state-of-the-art ViT-based method IELT [24], our model achieves the same performance with less computational cost and fewer parameters, indicating that our attention mechanism can be fully realized as a replacement for the original attention mechanism for FGVC tasks.

#### 4.4.4. Comparison of Computational Complexity

In contrast to the ViT model in Table 10 on one Nvidia GeForce RTX 3090 GPU, our approach is associated with a significant reduction in computation and training time. The table shows the number of parameters for both methods, the amount of computation to process an image, and the memory footprint and training time for ViT using batch=8 and DDA-Trans using batch=32 with FP32. It is worth noting that for our DDA-Trans model, the vast majority of the computation occurs in the query mappings, key mappings, value mappings, output mappings, and linear transformations in each layer of the FFN network and that the amount of computation in our linear attention is only 0.5 G. Our research focuses on solving the problem of the computational cost of attention computation, which is quadratically related to the image resolution, and it is clear that our approach is successful in solving this problem.

**Table 8:** Comparison experiments with other state-of-the-art methods on the Stanford Dogs dataset.

Method	Backbone	Input Resolution	Acc (%)
NTS-Net [68]	RestNet-50	448 × 448	87.5
CIN [69]	RestNet-101	448 × 448	87.6
FBSD [70]	RestNet-50	448 × 448	88.1
CAL [57]	RestNet-101	448 × 448	88.7
Cross-X [39]	RestNet-50	448 × 448	88.9
MRDMN [71]	RestNet-50	448 × 448	89.1
API-Net [38]	DenseNet-161	512 × 512	90.3
MSHQP [72]	RestNet-50	448 × 448	90.4
PRIS [66]	RestNet-101	448 × 448	90.7
ViT [42]	ViT-B_16	448 × 448	91.4
TransFG [44]	ViT-B_16	448 × 448	91.4
FFVT [45]	ViT-B_16	448 × 448	91.5
AFTrans [26]	ViT-B_16	448 × 448	91.6
IELT [24]	ViT-B_16	448 × 448	91.8
RAMS-Trans [28]	ViT-B_16	224 × 224	92.4
TPSKG [46]	ViT-B_16	448 × 448	92.5
ours	ViT-B_16	448 × 448	92.4

**Table 9:** Comparison experiments with other state-of-the-art methods on the NABirds dataset.

Method	Backbone	Input Resolution	Acc (%)
SCAPNet [62]	RestNet-50	224 × 224	82.8
Cross-X [39]	RestNet-50	448 × 448	86.4
HGNet [73]	RestNet-50	448 × 448	86.4
DSTL [74]	Inception-v3	560 × 560	87.9
PAIRS [75]	RestNet-50	448 × 448	87.9
GaRD [63]	RestNet-50	448 × 448	88.0
API-Net [38]	DenseNet-161	512 × 512	88.1
PRIS [66]	RestNet-101	448 × 448	88.4
CS-Part [76]	RestNet-50	448 × 448	88.5
MGE-CNN [67]	SENet-154	448 × 448	88.6
ViT [42]	ViT-B_16	448 × 448	89.9
TransFG [44]	ViT-B_16	448 × 448	89.9
TPSKG [46]	ViT-B_16	448 × 448	90.1
IELT [24]	ViT-B_16	448 × 448	90.8
ours	ViT-B_16	448 × 448	90.8

**Table 10:** Comparison of model computational complexity on the CUB-200-2011 dataset.

Method	Layer	Params	Flops	Batch	Memory	Time	Accuracy (%)
ViT	12	86.4 M	77.8 G	8	22.3 GB	9.5 h	90.8
ours	11	79.1 M	55.8 G	32	20.9 GB	2 h	91.7

### 4.5. Visualization

**Figure 8:**
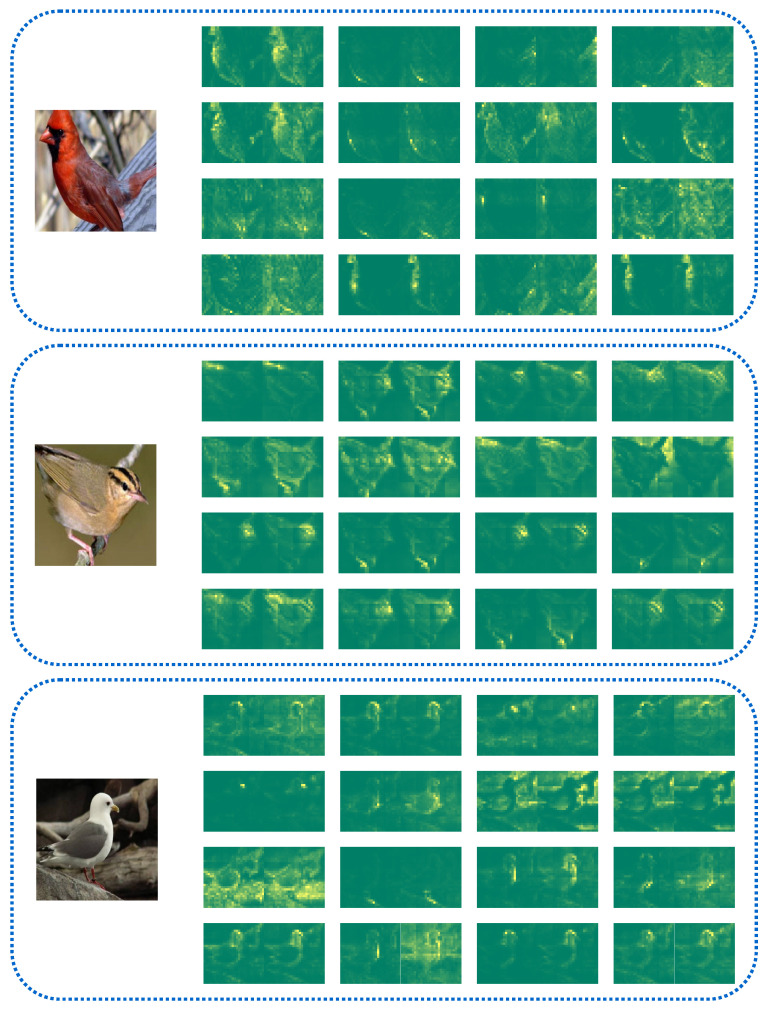
Visualization results of intralayer semantic-dependency attention. The left side of the figure is the original image, and each of the remaining two adjacent images is a group representing that they use the same central tokens, where the left image in each group represents the iteratively updated attention map of the center tokens, and the right image in each group represents the extracted attention map of the global tokens with respect to the center tokens, and all groups of images represent the information propagation visualization of all the center tokens within a given layer of the model within the semantic-dependency attention pathway.

We visualize the information interaction principle based on the center token in the model semantic-dependency pathway of one layer, as shown in Figure 8. We can see that all the center tokens capture the semantic information of foreground targets in the image with their own characteristics and complementarities and, at the same time, achieve the importance of distinguishing the difference between background noise and foreground targets for discriminative needs. In addition, the center tokens do not pay the same attention to the semantic information, namely, some focus on the whole outline, some choose to focus on some components, and some search for details, but all of them are successful in building the interaction paths of the tokens in the similar semantic space and modeling the semantic dependencies.

**Figure 9:**
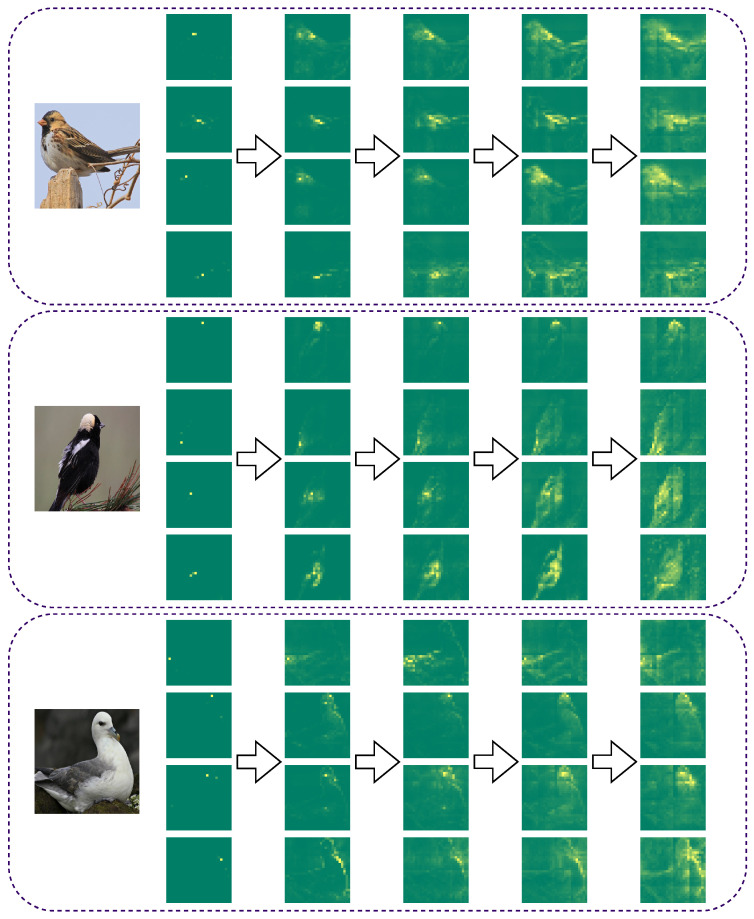
Visualization results for the distribution of center tokens capturing within the backbone network. The original image is shown on the left side of the figure, and the results of visualizing the updated attention weights of the selected center tokens in forward propagation are shown on the right side of the figure, where from left to right represents the network from shallow to deep.

In addition, we visualize center tokens in the backbone network refining the capture of semantically relevant tokens in iterative updates as shown in Figure 9. We can find that the center token has the ability to capture semantic clustering core tokens in the shallow network and then iteratively updates and grows the capture ability to semantically similar region tokens as the network forward propagation process continues to achieve complete dependency modeling of discriminative semantic tokens of foreground targets in the deep network; additionally, the visualization results fully demonstrate the working principle of the center token in the semantic-dependency attention pathway of the backbone network.

## 5. Conclusions

In this study, we propose a novel dual-dependency attention mechanism that decomposes the interaction modeling of global tokens into position-dependency grouped attention and semantic-dependency central attention and for the first time achieves linear computational complexity attention that can be directly used to replace the original attention mechanism for the FGVC task without the need for specific pre-training. Moreover, we design a knowledge-based discriminative ability enhancement module to improve the sensitivity of the model to high-confidence class-related discriminative cues. Combined with the above innovations, our approach successfully achieves a significant reduction in computational cost while demonstrating performance that rivals current state-of-the-art methods.

In the future, we will explore how to build more efficient fine-grained feature learning strategies and seek to promote this attention approach to a wider range of downstream tasks.

## Figures and Tables

**Table 1:** Three standard fine-grained visual classification datasets are used in our experiments.

Dataset	Class	Train	Test
CUB-200-2011 [54]	200	5994	5794
Stanford Dogs [55]	120	12,000	8580
NABirds [56]	555	23,929	24,633

**Table 2:** Ablation experiments on the impact of each part of our model on performance.

CTA	PDA	SDA	DAE	CUB-200-2011	Stanford Dogs	NABirds
✕	✓	✕	✕	89.4	89.9	88.5
✓	✕	✓	✕	90.6	91.0	89.7
✓	✓	✓	✕	91.2	91.8	90.3
✕	✓	✕	✓	90.1	90.4	89.1
✓	✕	✓	✓	91.1	91.6	90.2
✓	✓	✓	✓	91.7	92.4	90.8

**Table 3:** Ablation experiments on the impact of the hyperparameter on the CUB-200-2011 dataset.

Value of *m*	Acc (%)	Value of *g*	Acc (%)	Value of *t*	Acc (%)
8	91.3	24	91.4	4	91.5
12	91.5	48	91.7	6	91.6
16	91.7	96	91.7	8	91.7
20	91.6	192	91.8	10	91.5

**Table 4:** Ablation experiments on the impact of the center token aggregation method on model performance.

TSA	CTA	CUB-200-2011	Stanford Dogs	NABirds
✓	✕	91.1	91.7	89.9
✕	✓	91.7	92.4	90.8

**Table 5:** Ablation experiments on the impact of the attention method for the position-dependency pathway on model performance.

LDA	GSA	LDA and GSA	CUB-200-2011	Stanford Dogs	NABirds
✓	✕	✕	91.6	92.2	90.7
✕	✓	✕	91.5	92.1	90.6
✕	✕	✓	91.7	92.4	90.8

**Table 6:** Ablation experiments on the significance of center token interaction in the semantic-dependency pathway on model performance.

Center Token Interaction	CUB-200-2011	Stanford Dogs	NABirds
✓	91.3	91.8	90.2
✕	91.7	92.4	90.8

**Table 7:** Comparison experiments with other state-of-the-art methods on the CUB-200-2011 dataset.

Method	Backbone	Input Resolution	Acc (%)
FDL [58]	DenseNet-161	448 × 448	89.1
CSC-Net [59]	RestNet-50	224 × 224	89.2
DP-Net [60]	RestNet-50	448 × 448	89.3
MCEN [61]	RestNet-50	448 × 448	89.3
SCAPNet [62]	RestNet-50	224 × 224	89.5
GaRD [63]	RestNet-50	448 × 448	89.6
PMG [64]	RestNet-50	550 × 550	89.6
API-Net [38]	DenseNet-161	512 × 512	90.0
CPM [35]	GoogleNet	over 800	90.4
CAL [57]	RestNet-101	448 × 448	90.6
ViT [42]	ViT-B_16	448 × 448	90.8
TransIFC [65]	ViT-B_16	448 × 448	91.0
TransFG [44]	ViT-B_16	448 × 448	91.1
TPSKG [46]	ViT-B_16	448 × 448	91.3
RAMS-Trans [28]	ViT-B_16	448 × 448	91.3
FFVT [45]	ViT-B_16	448 × 448	91.4
DCAL [27]	ViT-B_16	448 × 448	91.4
SIM-Trans [25]	ViT-B_16	448 × 448	91.5
AFTrans [26]	ViT-B_16	448 × 448	91.5
IELT [24]	ViT-B_16	448 × 448	91.8
ours	ViT-B_16	448 × 448	91.7

## Data Availability

Data are contained within the article.
